# Phytoremediation of Pollutants: Applicability and Future Perspective

**DOI:** 10.3390/plants12132462

**Published:** 2023-06-27

**Authors:** Maria Luce Bartucca, Martina Cerri, Cinzia Forni

**Affiliations:** 1 Department of Agricultural, Food and Environmental Sciences, University of Perugia, Borgo XX Giugno 74, 06121 Perugia, Italy; marialucebartucca@gmail.com (M.L.B.); cerri.martina@gmail.com (M.C.); 2 Department of Biology, University of Rome Tor Vergata, Via della Ricerca Scientifica, 00133 Rome, Italy

Environmental pollution is a global issue since it is spreading worldwide, affecting entire ecosystems. Phytoremediation of pollutants is a renowned and environmentally friendly technique to extract or degrade several pollutants. Phytotechnologies are based on the ability of several plant species, and, to a certain extent, microalgae, to remediate soil, water, and air resources and to rehabilitate ecosystems. Over the years, phytoremediation has gained the favor of researchers and stakeholders, even though its application is still limited. In this Special Issue, different papers discuss several aspects and perspectives of this technique. The methodological approaches of phytoremediation are described in the review by Bartucca et al. [1], where biostimulants’ positive effects on the plants’ remediation activities have been reported. Several biostimulants of different origins (from bacteria and algae to fungi and plants) have been described and considered for improving phytoremediation activity, providing a broad spectrum of possible applications.

The efficiency of remediation depends mainly on plant species and the type and concentration of contaminants. Metals are among the most dangerous pollutants as they are not biodegradable. Metals tend to accumulate in soils, posing potential risks to surrounding ecosystems and human health. Basic principles, techniques, and potential anticipated prospects of phytoremediation of the metals, in addition to an overview of the biochemical aspects and the exertion of macrophytes in phytoremediation, have been provided by Sabreena and co-authors [2]. The remediation activity of different aquatic macrophytes species is discussed in this review.

Even though plants have shown considerable potentiality to uptake metals (phytoextraction) for in situ remediation, this technique still presents limitations and deserves further studies. Metal contamination of soil is also the focus of the review by Venegas-Rioseco and co-authors [3], which discusses the advantages and limitations of different strategies for enhancing HM accumulation and tolerance. Native plants, naturally growing on soils contaminated by metals, can be selected for phytoextraction and revegetation. Additionally, these plants, which colonize sites with high metal concentrations, can be the best candidates as a source of target genes to be used in genetic engineering research. Studies are reported on applying genetic engineering strategies (i.e., gene editing, stacking genes, transformation, and epigenetic regulation) to improve the plant phytoextraction potential. According to the authors, to enhance phytoextraction performance in metal-polluted soils, the best candidate genes are those related to metallothionein (MT), phytochelatin (PC), phytochelatin synthase (PCS), metal transporters, and antioxidant- activities. Legal and normative limitations have also been considered by the authors, who suggest further development of regulatory frameworks that should effectively drive such genetic engineering technologies to beneficial applications [3]. 

Petroleum is a major pollutant of ecosystems, and many bacteria were demonstrated to help its remediation. Kuzina and colleagues [4] focused on the effect of hydrocarbon-oxidizing auxin-producing bacteria on the growth, biochemical parameters, and hormonal status of barley plants in the presence of oil. They tested *Enterobacter* sp. UOM 3 and *Pseudomonas hunanensis* IB C7, finding that they could mitigate the negative effects of abiotic stress (caused by the oil) on plant growth. It is worth noting that the most substantial inhibitory effect of oil was detected in the spikes. *Enterobacter* had a higher positive effect on the length of the main spike, and number of spikelets per spike, compared to *P. hunanensis*. The authors also analyzed flavonoids and proline content, which are known to intervene in the adaptation to stressful environments, and the effects of oil pollution on the hormonal production of barley plants. Accumulation of IAA in shoots could, indeed, protect plants from stress factors by activating the antioxidant system; furthermore, since the presence of a pollutant reduces the availability of water and ions to plants, maintenance of the correct hormone balance to support good root growth is a vital plant response for adaptation to stressful condition. The data obtained indicate that introducing microorganisms weakened the negative effects of abiotic stress caused on barley plants by the presence of oil.

Yasseen and Al-Thani [5] elucidate the potential of endophytes’ use in lands contaminated by industrial wastewater and in the remediation of saline soils, focusing on the situation in Qatar. Halophytes, which in Qatar are mainly semi-woody shrubs, perennials, and succulents, are promising candidates for soil desalination and cleaning from toxic ions. The authors discussed all the mechanisms adopted by endophytic bacteria and fungi to support halophytes in the desalination of soils and phytoremediation of industrial wastewater, with many case studies mainly regarding *Bacillus* spp. and *Pseudomonas* spp. They concluded that their cooperation is an innovative approach that could promise to solve the pollution problems of soils and waters in an environmentally friendly way. Biotechnologies could increase the efficiency of remediation, and a monitoring system in recycling plants used for phytoremediation is also advocated.

As shown by previous studies, the intervention of microorganisms is a very relevant process in phytoremediation. Bioaugmentation is carried out by inoculating contaminated soil or water with pre-grown microbial cultures to improve the phytoremediation technology. Due to its strong potential in this field, this technique must be deepened and tested. The effect of the application of the commercial product RhizoVital^®^42, containing *Bacillus amyloliquefaciens* FZB42, on soil polluted by potentially toxic elements (PTEs), critical raw materials (CRMs, such as germanium -Ge), and rare earth elements (REEs) was the object of the study by Okoroafor et al. [6]. Results showed that *B. amyloliquefaciens* successfully integrated (relative abundance 1%) into the bacterial community, which was not altered by the enrichment. The inoculated soil was planted with *Zea mays* and *Fagopyrum esculentum*. The phytoremediation tests showed that *B. amyloliquefaciens* could either improve or reduce the assimilation of toxic elements and nutrients in the plant in a species-specific manner. In general, the inoculation enhanced the uptake of As, Cu, and other nutrients while decreasing the accumulation of Ge, Cr, and Fe. The results obtained in the study can be used in agricultural or environmental remediation projects.

Water pollution is one of the most severe problems concerning environmental issues. Therefore, innovative studies on phytoremediation should aim to broaden the knowledge of the techniques used to date and to develop further methodologies, considering the nature of new pollutants released in our waters. In this Special Issue, different studies have been addressed on the phytoremediation of polluted water and wastewater. Their central themes focus on the employment of algae and macrophytes (which play a major role in this area) and on optimizing the systems used for water cleanup.

A bibliometric study [7] based on the Scopus database revealed that in the years 2000–2020, the scientific community had increased its interest in the potential use of algae in the biodegradation of phenol. China, Spain, and the United States contributed the most significant proliferation of research on the topic. Phenols (phenol, cathecol, chlorophenol, bisphenol A, etc.) are primarily released into the environment by petroleum refineries, agricultural sources, and petrochemical, automotive, pharmaceutical, textile, and food and beverage industries. These compounds are highly toxic to humans and animals and are characterized by low biodegradability and high solubility in water. Since accidental spillage has caused rising phenol contamination in water, “phycoremediation” (a technique that uses algae to remediate polluted sites) is increasingly considered. In particular, *Chlorella* and *Scenedesmus* spp. have been demonstrated to efficiently convert phenols into less toxic derivatives. The process proceeds aerobically and is regulated by phenol-degrading enzymes, such as lignin peroxidase. 

Chiellini et al. have successfully proposed a novel approach to remediate wastewaters contaminated by cigarette butts (CB) by using microalgae [8]. In the study, six microalgae strains (one from the family of Scenedesmaceae, two *Chlamydomonas debaryana,* and one *Chlorella sorokiniana*) were exposed to CB wastewater with dilutions ranging from 1 to 25% (corresponding to 5 to 125 butts L^−1^). The analysis of microalgal physiological status revealed that photosynthetic pigment production was commonly inhibited in a concentration-dependent manner (generally, from a CB concentration ≥ 5%). Results from CB wastewater remediation test showed that the most abundant pollutant, nicotine (49.4%), was the most difficult to removal by microalgae. The multiple-factor analysis correlated the low ability to remove nicotine with the inhibition of chlorophylls, suggesting the detrimental effect of this alkaloid on photosynthetic pigments. On the other hand, benzonitrile (5.2%); 1,2,3-propanetriol, diacetate (4.0%); and the silicon (Si)-based compounds (33.5%) were entirely or almost completely removed by the microalgal strains. Unexpectedly, hydrocarbons and additives, such as plasticizers (5.2%), were mainly removed at the highest concentration of CB wastewater. After the study, the authors highlighted the high performance of *Chlamydomonas* strain in removing pollutants (69%) at 5% CB wastewater (corresponding to 25 butts L^−1^ or 5 g CB L^−1^) while maintaining its growth and pigments at control levels.

Most water phytoremediation studies carried out so far were conducted in closed systems. However, the use and development of continuous systems are crucial to the implementation of large-scale phytoremediation. The study by Sigcau et al. [9] focused on optimizing a continuous phytoremediative water treatment system involving the aquatic macrophyte *Lemna minor* in the removal of nitrogen (N). In detail, the purpose of the study was the online control of pH in the discharge water to use this parameter as the sole input variable of the system for the prediction of N removal. In fact, nitrate absorption by plants implies the simultaneous co-absorption of H^+^ ions and the release of OH^-^ ions in the growth medium, leading to water alkalization. At the same time, nitrate assimilation by plants is strictly related to biomass production. The study established the relationship among acid dosing (protons released to maintain the pH constant at 6.5), nitrate removed, and plant biomass production. The measure and control of the pH of the water medium in the system thus permitted to calculate and optimize the amount of nitrate absorbed (over 80% N removal rate in 7.2 L day^−1^), while maintaining a constant biomass layer of *Lemna* plants.

The study conducted by Tshithukhe et al. [10] aimed to assess the phytoremediative potential of native and non-native macrophyte plant species towards heavy metals (HM) removal from the Swartkops River (South Africa) water and sediments. Urban, agricultural, and industrial discharges in this site release high quantities of HMs (Zn, Fe, Cd, As, Cr, Pb, Hg, and Cu). Ten sites upstream and downstream of the phytoremediative plant mats were sampled. The plant analysis (bioconcentration factor-BCF) evidenced the selected species’ potential in the proposed objective. In particular, the free-floating non-native *Pontederia crassipes* showed the highest HMs assimilation potential, followed by the submerged native *Stuckenia pectinatus* and the three native emerged *Typha capensis*, *Cyperus sexangularis* and *Phragmites australis*. However, due to high variation among sites, the sampling results did not show a consistent decreasing trend in the water and sediment contamination along the river course. The authors concluded that the continuous contamination inputs along the riverside nullified the removal operated by macrophytes.

In the past 10–20 years, plant researchers have developed a sophisticated understanding of how plants responding to stress caused by exposure to pollutants alter their gene expression. However, plants alone are not always capable of effectively responding to heavy environmental contamination, and often phytoremediation efficiency, determined primarily on closed and controlled environments and on a single pollutant, is species specific. Undoubtedly, much work remains to be conducted before phytoremediation becomes a mainstay of agricultural and silvicultural practice to remove contaminants. To augment plant survival strategies and their remediation efficiency, we can apply microbial-assisted phytoremediation, biostimulants, or genetic engineering strategies. Recently, an operative handbook for eco-compatible remediation of degraded soils has been published [11]; it reports the experience carried out during five years of the Ecoremed project funded by the European Union and also provides operational suggestions, such as a more comprehensive utilization of phytostabilization approach.

Altogether, the different approaches may be used in a meaningful way to help plants not only to grow in polluted environments, but also to more efficiently phytoremediate the ecosystems.
